# Determinism and moral agency in *4 Ezra*

**DOI:** 10.1177/09518207251328592

**Published:** 2025-04-24

**Authors:** Dustin Barker

**Affiliations:** University of Toronto, Canada

**Keywords:** *4 Ezra*, apocalypticism, determinism, Deuteronomic pattern, free will, human moral responsibility

## Abstract

The significant consensus in contemporary scholarship on the connection between the message of *4 Ezra* and the figure of Ezra’s transformation in *4 Ezra* also seems to be accompanied by tendencies to contrast the rational against the experiential, and an implied incoherence in the way that the author articulates deterministic notions on the one hand and human moral responsibility on the other hand. I argue that the existential component of Ezra’s transformation is underlaid by the rational. To illustrate, I apply Lorenzo DiTommaso’s framing of Ezra’s “conversion” to the author’s articulation of determinism. Ezra’s pre-conversion deterministic thinking is rooted in the Deuteronomic pattern whereas Ezra’s post-conversion determinism is thoroughly apocalyptic. A rational coherence underscores the author’s articulation of human moral responsibility and the latter view. The rational coherence of these two elements is essential for the author’s goal of effecting conversion and frames moral action as a symbolic performance of collective identity for the text’s implied community.

The transformation of the figure of Ezra is the message of *4 Ezra*. I contend that this transformation should be understood in both rational and existential terms and that its rational import can be seen in the way in which the author presents a positive and coherent case for apocalyptic determinism. This intervention is rendered necessary by a tendency in contemporary scholarship to emphasize the existential elements of Ezra’s transformation in contrast to the rational. Indeed, for some scholars, it is not at all clear how *4 Ezra* provides rationally coherent answers to the problems that it raises.

Michael E. Stone’s argument for the centrality of “the Odyssey of Ezra’s soul”^1^ has proven to be foundational for subsequent scholarship in locating the work’s unified vision and message within its depiction of Ezra’s metamorphosis.^2^ There is now a consensus in scholarship concerning the centrality of Ezra’s conversion. It would be a mistake, however, to understand this consensus as an agreement on precisely how this consolation is depicted or what exactly this consolation represents. To cite but a few examples, Stone understands the consolation that Ezra experiences as “a major religious experience, a conversion . . . That which had been outside, as ‘God’ or ‘the angel,’ become[s] dominant.”^3^ Bruce Longenecker argues that Ezra’s conversion represents a program of healing for a national ethos which is achieved “not by the silencing of confusion and bewilderment, nor by the repression of pain or sorrow, but by expressing such sentiments fully, in order that they might be managed, healed, and transcended.”^4^ Karina Martin Hogan understands Ezra’s conversion so that apocalypticism resolves a debate between two different wisdom traditions. Moreover, this apocalyptic solution represents a non-rational resolution to a problem that proves to be irresolvable on rational grounds.^5^ Lorenzo DiTommaso, contra Hogan and building on Stone’s view, describes Ezra’s conversion as a movement from “the Deuteronomic worldview to the apocalyptic.”^6^ He also extends Ezra’s conversion experience to the text’s social function; “The author wishes that his readers undertake the same existential journey as he once did.”^7^ Hindy Najman understands Ezra as the creation of an exemplar for the reader. “By identifying with Ezra, the reader undergoes formation as a subject capable of continuing a covenantal life in the wake of destruction.”^8^ She argues that this covenantal life is reoriented away from rebuilding the temple and is focused instead on the future: “the divine revelation of the Temple and the imminence of the world to come.”^9^ Such focus facilitates the reader’s learning of how to mourn and lament the destruction of Jerusalem and their task of obeying in the present, where the scriptures have been reconfigured and revitalized for the community.^10^ Lydia Gore-Jones presents the message of *4 Ezra* in terms of a concern for Israel’s covenant and election in contrast to a more abstract notion of theodicy.^11^ The solution, she proposes, is “the adoption and promotion of the eschatological worldview, instead of the Deuteronomic worldview.”^12^ Yet, she also argues that “the author does not abandon the old; even in the new scheme of eschatological judgement the Deuteronomic notion of Torah obedience plays a decisive role in a soul’s afterlife.”^13^

Of these different constructions of Ezra’s consolation, there is a noticeable tendency for drawing a dichotomy between what are understood to be “rational solutions” versus “emotional” or “existential” solutions. Already in 1977, Albert Thompson argued that *4 Ezra* is a theodicy that does not offer rational solutions.^14^ Instead, it offers an experiential solution.^15^ Hogan explicitly portrays the work’s apocalyptic resolution as “non-rational” in contrast to the two “rational” sapiential approaches that are ultimately rejected.^16^ Dereck Daschke contrasts the “non-verbal” apocalyptic resolution to the dialogue sections where Ezra, Uriel, or God can get the upper hand.^17^ He writes that

the transformation in 4 Ezra occurs not by some cognitive epiphany but rather in the course of a wrenchingly sad narrative, punctuated by a glorious vision, affirms what the incomplete disposition of the dialogues suggests. There is no compelling argument to justify the gap between traditional expectations and the world as it is.^18^

For Longenecker, Ezra’s transformed state is characterized by Ezra’s refusal “to allow his sorrow to deteriorate into doubts concerning God’s sovereignty or righteousness; here, remorse does not degenerate into complaint and accusation against God.”^19^ While Longenecker does not deny an intellectual dimension within *4 Ezra*,^20^ his emphasis on putting a stopper on grief implies that there is no rational resolution to the problem that causes Ezra’s grief.^21^ Similarly, Najman affirms that Ezra is offered rational responses to his inquiries; and yet, this cannot be satisfactory. Emotional resolution is not interwoven with rational resolution; it is layered on top of and beyond it.^22^ Katell Berthelot likewise affirms a conceptual continuity of content between Uriel’s discourse in the dialogues and the work’s concluding visions. But she also emphasizes that the dialogues’ initial teachings are insufficient to persuade Ezra. Ultimately, “only a direct revelation of God—or of God’s ways—is a satisfactory answer to the problem of evil. The answer is not rational, but existential.”^23^ Gore-Jones does argue for what might be framed as a rational solution, but her framing of the solution itself reflects an implied, if unacknowledged, incoherence. She writes that the apocalyptic solution in *4 Ezra* is not meant to “abandon the Deuteronomic tradition enshrined in the name of Moses.”^24^ But this claim fundamentally misunderstands both apocalypticism as a worldview, according to DiTommaso,^25^ and what George Nickelsburg has articulated in terms of the Deuteronomic pattern.^26^ Such a view, it seems to me, introduces a measure of conceptual incoherence into the perspective that is affirmed within *4 Ezra*.

A measure of incoherence on the part of the author, suspected or at least implied, becomes apparent in places where scholarship addresses notions of predeterminism within *4 Ezra.* For example, in a brief comment in his 1979 commentary, Michael Knibb juxtaposes the author’s predetermined historiography against the view that humans are also responsible for their own destiny.^27^ Stone notes that the view of an evil inclination as a human weakness inherited from Adam stands in tension with the notion of “free-will” that frequently arises in the work.^28^ He also affirms that, for the author of *4 Ezra*, “the idea of free will is so deeply rooted that he does not work out the ramifications of his predestinarian assumptions.”^29^ Hogan notes “a tension between free will and determinism throughout Uriel’s speeches,”^30^ such that Uriel contrasts God’s implicit foreknowledge of the righteous against repeated stress that “keeping the law is a matter of free choice and that all people have the ability to do so.”^31^ Jonathan Moo writes that “the author of *4 Ezra* does not seem concerned to reconcile such apparently contradictory statements regarding divine sovereignty and human responsibility, allowing the same speaker to emphasize either or both as the context warrants.”^32^ Najman writes that *4 Ezra* “neither affirms nor denies that punishment is still being exacted for Adam’s sin, and it neither affirms nor denies that human beings have free will.”^33^ Regarding humanity’s universal grappling with the evil heart and the reality that some individuals are in fact obedient, she states that “whether that is because of human choice or divine election, we are not told.”^34^ It is clear, however, that *4 Ezra* does make statements that could support either position. As such, Najman’s statement is perhaps less the result of textual silence and more the result of what is perceived to be a lack of coherence between a dichotomous construction of human choice and divine election.

I do not disagree with the contention that *4 Ezra* contains a significant existential and emotional component. Indeed, I would not argue against those scholars who understand *4 Ezra* to present this existential element as a necessary component that complements its rational element. That is, *4 Ezra* provides a resolution that is more, but indeed not less, than rational. However, as I have just outlined, the existential tends to be framed against the rational in scholarship. I therefore contend that the rational and the experiential are entangled in Ezra’s experience of comfort. While Ezra’s consolation involves a significant experiential component, in terms of the narrative, it is also true that Ezra’s consolation comes only after he has internalized Uriel’s thought.^35^ The existential component of Ezra’s relief proceeds from an internalized rational shift.

I argue that this rational element is coherently expressed in precisely that area that, as was seen above, contemporary scholarship has associated with incoherence; namely, the author’s perspective on determinism and human moral responsibility. An interesting feature of *4 Ezra* is that Ezra’s thinking remains deterministic in both his pre-conversion distress and post-conversion relief.^36^ While this observation might support the view that Ezra’s comfort is essentially irrational, a closer look at Ezra’s deterministic thinking reveals that his pre-conversion and post-conversion modes of thought are not deterministic in precisely the same way. I propose that DiTommaso’s contention concerning Ezra’s conversion, that it represents a shift from a Deuteronomic worldview to apocalypticism,^37^ may be fruitfully applied in an analysis of Ezra’s pre- and post-conversion modes of deterministic thinking. To argue this, I compare Ezra’s deterministic thinking in both his pre-conversion and post-conversion modes of thought. I examine how the former is both grounded in and becomes a critique of the Deuteronomic pattern, and how the latter is thoroughly framed by apocalypticism. Moreover, I examine how human moral responsibility is conceptualized within the author’s affirmative apocalyptic worldview. In doing so, I will contend that the author’s deterministic apocalyptic historiography is indeed rationally coherent with their conceptualization of human moral responsibility. In contrast to a tendency in scholarship to frame the emotional and existential component of *4 Ezra* against the rational, and although the consolation that apocalypticism proffers to both the figure of Ezra and the reader is indeed existential, it is not for this reason any less than rational. Ezra does receive coherent answers in response to his distress.^38^

## Ezra’s pre-conversion determinism

To argue that Ezra’s pre-conversion thinking is a determinism that, perhaps ironically, flows from and becomes a critique of the Deuteronomic pattern, I will first define what I mean by the “Deuteronomic pattern” and “determinism.”

### The deuteronomic pattern

George Nickelsburg identified the Deuteronomic pattern (DP) in reference to second temple texts.^39^ It is a theological historiography^40^ framed by the conceptual touchpoints of election, covenant, reward/punishment, and resolution.^41^ In this view, because God’s acts in history in response to Israel’s covenantal conduct, human agency is the mechanism that drives history forward. As such, history is conceptualized as a cyclical pattern of obedience, disobedience, and restoration, where the nation’s fortunes change in accordance with its conduct.^42^ When present circumstances are interpreted through the lens of this historiography, in circumstances of suffering, a past history of sin is entailed. In covenantal fidelity, future prosperity is expected. The distant future remains alterable.^43^

### Determinism

Bernard Berofsky observes that every deterministic system involves the notion that “at any particular time *t*, what occurs in the world at *t—*or, perhaps, what occurs at or prior to *t*—restricts the future possibilities to one.”^44^ While Berofsky’s inclusion of a causal connection between the present and future state excludes deterministic systems that understand the unalterable future state in terms of God’s intervention such that “normal” causal processes might be overridden, I think that he has identified what is common to all deterministic systems of thought. At its most basic level, deterministic thinking rejects a real possibility for alternate states of affairs. The future exists only as a possibility of one. When one provides reasons for this denial, specific modes of causality are identified.^45^ My investigation of determinism in *4 Ezra* focuses on those texts that I understand as denying a real possibility for alternate states of affairs.

### Ezra’s pre-conversion thinking and the DP

Ezra’s distress concerns God’s justice relative to Israel’s suffering and current national status.^46^ Importantly, Ezra’s complaints are grounded in the DP. For the sake of brevity, I examine one example as representative.

The essence of Ezra’s first complaint is found in 3:28–36.^47^ He reflects upon Israel’s exile in Babylon, and after comparing the two nations, Ezra questions the fairness of the covenant. Israel is being punished for disobedience, but God tolerates the wickedness of Babylon.^48^ The DP grounds Ezra’s complaint in three ways. First, it assumes a distinct relationship between God and Israel by which Israel enjoys privilege and duty (that is, election and covenant). Second, for Ezra, Israel’s present suffering entails a history of sin because suffering follows disobedience (that is, reward/punishment). The present gives meaning to the past through an interpretive grid of covenantal conduct and consequence. Third, Ezra assumes that God governs the fate of Israel in response to the nation’s conduct. Had the nation acted differently, the nation’s present circumstances would accordingly differ. Here, human action is history’s driving mechanism.

Ezra does not question the truth of the points above. Instead, assuming their truth, he questions how the present reality is just. It is not fair that God punishes Israel through other nations or that God tolerates the wickedness of other nations because only Israel relates to God through covenant (3:32–36). In sum, the DP contextualizes Ezra’s first complaint and represents how the DP provides a framework for Ezra’s complaints up until his conversion.^49^

### Ezra’s pre-conversion determinism

Within the framework of the DP, Ezra articulates distress concerning his view that human action is predetermined (3:20–27; 7:45–48; 7:66–69; 7:116–126; see also 8:35–36; 8:42–45; and 9:32–37). In these passages, Ezra’s view of predetermined human action is consistent: (1) Adam’s sin involves a tragic inheritance for humanity such that (2) all humans possess an evil heart,^50^ which (3) results in an inability to avoid transgression.^51^

In Ezra’s pre-conversion mode of thinking, this view of predetermined human failing is problematic because, according to the DP, human action functions as history’s driving mechanism. On Ezra’s account, as reward and punishment are predicated upon human conduct, the covenant can guarantee only retribution because the evil heart conditions human action toward sin (cf. 8:35–36).^52^ In this way, then, Ezra’s pre-conversion deterministic thinking both flows out from and serves to drive his critique of the DP. It flows out from the DP because it retains human action as history’s driving mechanism. It critiques the DP on precisely the same basis. Because human action is history’s driving mechanism, the covenant can only guarantee retribution. Indeed, despite Ezra’s distress at the “evil heart,” he still assumes that human beings are moral agents who are accountable to God for their actions (cf. 8:35). Accordingly, Carol Newsom describes Ezra’s anguish in 7:62–68 in terms of “a consciousness that perceives both its moral responsibility and its inability to fulfill this responsibility.”^53^ So, too, Miryam Brand writes that

the author of *4 Ezra* describes the particularly cruel predicament of the human: while the law does not effectively combat the evil inclination from Ezra’s human perspective, it does make the hapless human a full moral agent in the eyes of God.^54^

Ezra views the DP to be bankrupt as a system because, in his pre-conversion account of predetermined human action where sin is unavoidable, God’s promises are contingent on Israel’s moral conduct. As a result, Ezra argues, God’s promises avail Israel nothing. On this basis, then, Ezra argues that “there is no redemptive corrective in the covenant.”^55^ For Ezra, God’s justice is faulty.^56^

And yet, as Ezra’s distress is resolved, his thinking continues to be framed by a thoroughly deterministic historiography. Although this observation might be made in support of an emphasis on Ezra’s consolation as being fundamentally irrational in nature, I contend that a closer examination of Ezra’s post-conversion thought reveals a distinctly different kind of determinism. By the time Ezra encounters the weeping-woman city, Ezra’s thinking has shifted from a determinism that is grounded in the DP to the apocalyptic determinism articulated by Uriel/God. Therefore, Ezra’s consolation is not simply related to an existential experience, but an experience grounded in a correlated shift in thinking.

## Apocalyptic determinism

Ezra’s post-conversion deterministic thinking is fundamentally distinct from his pre-conversion mode of thought by its apocalyptic framing. To argue this, I examine how the author’s affirmative determinism is articulated primarily through the voice of Uriel. However, to establish whether or not this view is properly apocalyptic, I must first define apocalypticism as a worldview.

### Apocalypticism

I follow Lorenzo DiTommaso in understanding apocalypticism in terms of a worldview.^57^ He contends that apocalypticism is a “suite of axiomatic propositions on the nature of time, space, and human existence” that are integrally related and unintelligible apart from the others.^58^ He describes these propositions, in their ensemble, as follows:

Apocalypticism asserts that a transcendent reality, concealed from casual observation yet operative on a grand scale, defines and informs existence beyond human understanding and the normal pale of worldly experience. It reveals a cosmos that is structured by two forces, good and evil, which have been in conflict since the dawn of history. It discloses the necessity and imminence of the final resolution of the conflict at the end of time, and the truth about human destiny.^59^

As a historiography, apocalypticism is oriented by a transtemporal eschatological horizon.^60^ “Apocalyptic time,” DiTommaso contends, “is linear, unidirectional, and finite.”^61^ Temporal finitude is understood in terms of God’s eschatological intervention, which brings “time and history to a climax.”^62^ Here, divine justice is reconceptualized as about the world to come, “beyond the limits of human history.”^63^ Accordingly, reward and punishment are re-envisioned as post-historical or post-mortem realities.^64^ Present circumstances no longer entail a specific history, as is the case within the DP. Rather, God’s end-time intervention sets the agenda for time’s linear and unidirectional progression.^65^ The end operates as history’s *telos* such that the divine plan, which dictates this end, functions as history’s driving mechanism.^66^ In this outlook, the future is not alterable.^67^

### Ezra’s post-conversion determinism

The author’s affirmative deterministic thinking articulated through the voice of Uriel, God, and the post-conversion Ezra is fundamentally deterministic in orientation. This is clear from how the future is presented as only a possibility of one. Thus, for example, the certainty of future events is communicated through the specificity of their detail, formulaic phrases,^68^ and a preference for the future indicative in a way that leaves no room for alternatives.^69^ Moreover, where the divine voice articulates a pre-creation-event perspective, such that “the end”^70^ is portrayed as having been planned before the act of creation (cf. 7:70), conceptually, “the future” concerns all that proceeds from God’s intention before the creation-event. That is, because “the end” was planned before human beginnings, the course of human history has only ever had a singular path that leads to a singular future. Indeed, the author’s deterministic historiography is neatly summarized toward the end of the work, where Ezra praises God for “the wonders that he does from time to time, and because *he governs the times and whatever things come to pass in their seasons*” (13:57–58; emphasis added).

The certainty of the future is further reinforced by how Ezra receives the interpretation of his dream-visions after his encounter with the weeping woman-city. They are interpreted with divine authority by both God and the angel Uriel.^71^ Here, Ezra does not question their veracity. Rather, he seeks clarity.^72^ This dynamic underscores the binary epistemology referenced earlier in the work: “those who inhabit the earth can understand only what is on the earth, and he who is above the heavens can understand what is above the height of the heavens” (4:21).^73^ For the author, the future exists only as a possibility of one.

### The divine “plan”

The deterministic elements of the author’s thought are centered upon the notion of a “divine plan,” although it is not expressed in so succinct a term.^74^ Human history may be qualified as a “plan” because it is conceptualized in a manner that is intentional, ordered, and planned. Because God is explicitly identified as the source of the intent that guides this plan, it is also aptly qualified as “divine.”

The sense that history proceeds in an ordered and intentional manner, in that it is predestined in its major processes and great events, is conveyed through the following elements: (1) the periodization of time; (2) the present *saeculum* functions according to a given set of parameters; (3) a specific intent underlies the creation-event, “the end,” and the present and future *saecula*; and (4) “the end” supplies history with a particular *telos*.

First, history is schematized into predetermined periods of time. This is most noticeable in the later visions of *4 Ezra*. Here, time is periodized into a four-kingdom motif (11:39–40; 12:10–12), predetermined numbers of kings (12:16-22), and, with particular reference to the present *saeculum*, into twelve predetermined parts (14:11–12).^75^ This schematization of time imbues history with a sense of order and intention such that it progresses along a predetermined chronology. This sense of order and intention is further reinforced by passages like 7:74, where God’s patience with humanity is not for humanity’s sake, but “because of the time that [God] has foreordained.”^76^ Similarly, just as time is conceptualized as predetermined numerical sets, so too, both the righteous who will be saved and the present *saeculum* are conceptualized as predetermined measures that must be balanced before God’s intervention (4:35–37).

Second, passages such as 4:28–32 and 5:44–47 portray the present *saeculum* as functioning within a given set of parameters. The first passage describes evil in the world as proceeding from Adam and necessitating a harvest, like a seed that is sown. As a result, cause and effect take on a moral quality: good and evil actions create unavoidable effects that follow the moral quality of their cause—the present *saeculum* is not simply ordered but follows a particular moral ordering. The second passage likens the world to a woman’s womb, which cannot produce offspring contrary to its natural capacity. The world is organized with particular natural capacities that cannot be altered before the eschaton.^77^ These two passages thus convey the overall sense that God has made the world to function in an orderly and unalterable manner before the end-time judgment (cf. 6:11–20).

Third, the author highlights a specific intention and purpose in the creation-event. For example, at 6:1–6 (cf. 7:10–16; 7:70), Uriel lists a series of phenomena before which (*antequam*) God planned (*cogitaui*) the creation such that all of creation can *only* be attributable to God’s intent and power.^78^ In context, Uriel underscores God’s unique agency and intention at creation to provide a “just as, therefore” kind of argument regarding God’s end-time visitation to creation.^79^ Just as the creation illustrates the unopposable potency of God’s intention and agency, the end of God’s visitation will also be characterized by the same quality of God’s agency; God’s agency guarantees its realization. Moreover, the effect of highlighting God’s agency and intention at the juxtaposition of the beginning and end of the world is also to include all things in between. The intent or plan that underlies creation, effected at the beginning and still operative at “the end,” entails its activity during the interim.

Elsewhere, Uriel emphasizes that both the present and future *saecula* are imbued with this same sense of intent and purpose. At 7:10–16, Uriel describes the nature of the present world as a kind of trial whereby the “living” (*qui uiuunt*; 7:14) receive their inheritance by passing through suffering (*angusta et uana haec*; 7:14; cf. 7:127; 8:2).^80^ But Uriel also affirms that a future *saeculum* has been created specifically for the righteous. Indeed, “the Most High has made not one world but two” (7:50).^81^ Both the present and future *saecula* are, therefore, imbued with a sense of intent and purpose. In this present *saeculum*, the righteous must suffer before inheriting the future *saeculum*, which has been intended for them from the beginning (7:50; 8:1). The intent that underlies the creation-event, “the end,” and the two *saecula* reinforces the notion that history is both planned and ordered.

Fourth, in passages like 7:70 (cf. 6:1–6; 9:4), the “end” is portrayed as planned or intended prior to the creation. Concerning this verse, Stone comments that here, “the word ‘prepared’ is to be noted as a technical term describing the precreation of the eschatological things.”^82^ This “end,” then, clearly belongs to a transtemporal and transhistorical context. It not only preexists history but also represents the end of the present historical *saeculum* and a transition to the future *saeculum* (cf. 7:50; 14:34–36).^83^ Conceptually, the author locates the transtemporal “end” in a causal and determinative relation to the creation-event. In other words, much like a set of blueprints determines the way in which construction must proceed, God planned out the “end” prior to the creation-event. In turn, this “end” determines the beginning and keeps history progressing along an unalterable trajectory toward its realization. Thus, the “end” is an omni-orienting transtemporal, eschatological horizon within *4 Ezra*. Accordingly, it supplies history with a particular *telos*, inexorably pulling history forward along a singular path toward its realization.

In sum, the author of 4 Ezra portrays history as a series of ordered, intentional, and orchestrated events. As a result, the author’s deterministic historiography may be aptly qualified as a “plan.” Where the future is conceptualized as a possibility of one, God’s plan serves as its animating *telos* where the transtemporal “end” serves as an omni-orienting eschatological horizon.

### The “divine” plan

That this “plan” may also be qualified as “divine” is relatively straightforward. God is regularly identified as the agency responsible for the intent and purpose which underlies history. While many passages could be cited, one particularly represents the author’s thought. At 6:1–6, God (through Uriel’s voice) emphatically highlights that creation can *only* be attributable to God’s intent and agency.^84^ God’s unique agency and intent are highlighted here in service to the author’s point concerning God’s end-time visitation.^85^ Just as creation is realized by God’s singular and unique agency, God’s end-time visitation will be realized in the same way such that God’s singular agency acts as a guarantee.^86^ As mentioned previously, the effect of highlighting God’s agency and intention at the juxtaposition of the beginning and end of the world is to also include all things in-between. In other words, where history is portrayed as intended, purposeful, and planned, God is identified explicitly as the source of this intent, purpose, and plan.

In this way, then, where the author’s deterministic historiography may be qualified as a “plan,” it may also be qualified as “divine.” Thus, God’s own intent and agency are understood to animate and empower the plan that provides history with its animating *telos*.

### Apocalyptic determinism

Throughout the author’s articulation of their affirmative determinism *via* the voice of Uriel/God, articulated in terms of the “divine plan,” it becomes clear that apocalypticism’s notional architecture thoroughly contextualizes this deterministic thinking. Conceptually, dualism pervades its temporality, epistemology, and sociology. The present *saeculum* is contrasted to the future *saeculum* so that the latter represents a clear break with the former. Indeed, God “has made not one world but two” (7:50). Heavenly knowledge is contrasted to earthly or mundane knowledge, implying both a spatial and ontological dualism (cf. 4:5–21; 5:38).^87^ Humanity is an antithetical collective binary of “the righteous” and “the wicked.” This binary division begins with Adam’s transgression (4:28–32), is maintained through the characterization of this world as a moral trial (7:9–14; 7:127–131), and is guaranteed by God’s judgment at the end of history where a righteous few transcend death in contrast to the torment of the wicked multitude (cf. 7:50; 7:59–99; 14:34–36).^88^ No alternatives to this definitional binary, neither in terms of trajectory nor end, are considered possible.^89^ In other words, the sociology of the unalterable future is conceptualized collectively. There are only two possible collective destinies in which the individual may participate.

Moreover, history’s trajectory is itself unalterable precisely because “the end” orients everything (cf. 6:1–6; 7:10–16; 7:70). This transtemporal, eschatological horizon supplies history with a teleological force such that the divine plan, and not human agency, functions as history’s driving mechanism. Indeed, God “governs the times and whatever comes to pass in their seasons” (13:57–58). As a result, the author’s affirmative deterministic thinking should be identified with the apocalyptic worldview; the determinism affirmed within *4 Ezra* is specifically an apocalyptic determinism.

## Human moral agency

Ezra’s pre-conversion distress is not simply connected to his deterministic view of history per se but particularly to how it relates to human action and moral agency. Ezra’s understanding is that humans in general, and Israel in particular, cannot avoid transgression. However, because the DP understanding of the covenant keeps human action at the center of its historiographical dynamics, it is designed to fail. For Ezra, where the covenant functions to explain the past and project future trajectories solely in terms of reward or punishment, punishment is the only logical outcome. As a result, Ezra feels that God’s promises are bankrupt and that God’s justice is impugned.^90^

Given both the intensity and coherence with which Ezra articulates his pre-conversion grief, it is doubtful whether an existential experience is enough to overcome the cognitive dissonance that Ezra articulates.^91^ Ezra’s initial critiques of God’s justice require that the author’s affirmation of apocalyptic determinism not continue to invalidate God’s justice by what it entails for human moral agency. Indeed, the care with which the author constructs both Ezra’s protests to God’s justice and Uriel’s answers, in my view, militates against reading the dialogues as an argument for an essentially irrational apocalyptic consolation. As a result, it is necessary to examine how human agency is conceptualized within the context of the author’s apocalyptic determinism, especially in light of the tension with which modern readers perceive these two elements.

Indeed, deterministic thinking generally entails some correlated view of human action and moral agency. In contemporary philosophical discourse, determinism is generally framed in stark opposition to notions of free will.^92^ Here, notions of free will are often conceptualized in terms of a distinct human faculty, that is, the will. Freedom constitutes the ability to choose or do otherwise in any given choice or action.^93^ When placed in the same circumstances of a particular choice or decision, one is free to the degree that one can do or choose other than they did. However, both Sophie Botros^94^ and Mladen Popović,^95^ on Stoicism and Second Temple Judaism, respectively, have argued against reading this contemporary philosophical discourse of determinism and free will onto ancient texts.

To examine the author’s conceptualization of human agency, I focus on how the author conceptualizes human moral responsibility. On what basis does the author predicate human culpability? In doing so, I follow the recommendations of Botros and Popović, avoiding terms like “free will” to prevent importing contemporary philosophical categories into the author’s account.

7:72–73 is one of the most explicit passages that addresses the issue of human moral agency within *4 Ezra*. It is representative of other relevant passages within *4 Ezra*:^96^

For this reason, therefore, those who live on earth shall be tormented, because though they had understanding, they committed iniquity; and though they received the commandments, they did not keep them; and though they obtained the law, they dealt unfaithfully with what they received.^97^ What, then, will they have to say in the judgment, or how will they answer in the last times? How long the Most High has been patient with those who inhabit the world!—and not for their sake, but because of the times that he has foreordained.

This passage articulates three elements that reflect the author’s conceptualization of human moral agency. First, moral agency is primarily concerned with action; people are judged for their actions because they are considered the source of their actions. In addition, humans understand God’s commands. Humans are accountable because they know the moral framework that qualifies action. Finally, humans are accountable because of intent. Reception of God’s commandments/law is contrasted with not keeping them and dealing with them unfaithfully. This characterization of behavior that is fraudulent, deceptive, or cheating (*fraudauerunt*) suggests something that goes beyond simple failure. The wickedness performed was not simply a failure to do good but an intention to do wickedness.^98^ *4 Ezra* thus frames human moral agency regarding action, understanding, and intent. In other words, human moral agency is conceptualized as *self-authored*, *self-propelled*, and *informed action*.

This does not, however, answer the question of how the author’s determinism impacts human action. 9:17–22 provides two additional nuances. God seems to employ a policy of non-intervention toward the wicked before the eschaton (9:22; cf. 3:8). Conversely, God exercises a positive agency for the righteous. With great difficulty (*uix ualde*), God

spared some . . . and saved for [himself] one grape out of a cluster. So let the multitude perish that has been born in vain, but let my grape and my plant be saved, because with much labor I have perfected them. (9:21–22)^99^

Here, the respective fates of the righteous and the wicked are not only characterized by the moral quality of their respective agencies but also by God’s inaction relative to the wicked in this *saeculum*, and some underdefined positive agency expressed by God toward the righteous. That this positive agency is not simply bestowed in terms of merit is indicated by the expression of this agency in terms of divine labor and other texts that reference righteous human characteristics that cannot simply be reduced to merit: qualities such as faith, repentance, and humility (8:49; 9:7; 9:11).^100^

This expression of a positive agency echoes what Ezra was previously told concerning eschatological realities for the righteous:

the root of evil is sealed up from you,^101^ illness is banished from you, and death is hidden; Hades has fled and corruption has been forgotten; sorrows have passed away, and in the end the treasure of immortality is made manifest. (8:53–54)^102^

The righteous who experience a sealing of the evil root in the eschaton have both worked hard to overcome such a root (7:92), and God has labored with them to perfect them (9:21–22). As such, the difference between “the righteous” and “the wicked” is in some sense established by God’s distinct, and yet underdefined, approach to the two groups.

## Summary

In order to examine the issue of rational coherence in relation to the way in which the author articulates both their apocalyptic determinism and notion of human moral responsibility, I break down the elements outlined above into clear summative propositions. In doing so, I recognize that these formulations are not the precise terms in which the author has expressed their own thought. I do, however, contend that they are accurate notional representations of what the author has articulated.

The author’s apocalyptic determinism and their conceptualization of human moral agency can be summarized as follows:

1. The divine plan, driven by God’s intent and agency and animated by the teleological force supplied by “the end,” is history’s driving mechanism. It reduces the future, as defined at any time, to the possibility of one.
2. The divine plan collectively determines human destiny, where humanity is binarily defined as “the righteous” and “the wicked.” In the transtemporal, eschatological “end,” “the righteous” transcend death while “the wicked” are tormented. The final tally of each group has already been determined.
3. The individual can participate in only one of these two collective destinies.
4. Human action may be predisposed to wickedness because of the evil heart.^103^ This predisposition is mitigated, in an undefined way, by God’s perfective labor on behalf of the righteous. God does not intervene to help the wicked.
5. Individual destiny is conceptualized as befitting one’s moral quality as it is reflected through personal action.
6. Human moral agency is conceptualized as self-authored, self-propelled, and informed action.

In comparing the above propositions one to another, there are no inherent logical contradictions among them. In other words, because they cohere together, it is reasonable to affirm that while apocalyptic determinism and human moral responsibility might appear to stand in tension within *4 Ezra*, a fundamental coherence underlies them. This should clarify some aspects of the tension that are sometimes found between those portions of *4 Ezra* that affirm deterministic thinking and other portions that affirm human moral responsibility. Where Ezra communicates a measure of cognitive dissonance concerning the notion of predetermined human action and God’s justice in his pre-conversion thinking, he articulates a determinism grounded in the DP where human agency is history’s driving mechanism. By contrast, the author’s affirmation of apocalyptic determinism with its emphasis on the divine plan is distinguished by an underlying rational coherence with its correlated affirmation of human moral responsibility.

With this analysis, I am not arguing that the author or their implied community would have understood such coherence in precisely this way. More likely, they would have affirmed that human moral responsibility and an exhaustively determined future are coherent within the context of what I have identified as apocalypticism. Such an exercise might be especially helpful for the modern reader. Where scholarship characterizes the author’s view in terms of determinism and free will, an implied incoherence is suggested because, in both contemporary philosophical and popular discourse, such terms are understood in mutually incompatible terms.^104^ As a result, I suggest two things. First, in *4 Ezra*, an effort must be made to distinguish between Ezra’s pre- and post-conversion modes of thinking. Second, to avoid a suggestion of incoherence where an underlying coherence may prevail, I think that the term “free will” should be abandoned in our analysis of ancient texts such as *4 Ezra*.^105^

However, this summary raises two other important questions, especially for the modern reader for whom determinism and free will are frequently understood to be incompatible. First, to what end is individual choice efficacious? Second, in keeping with the author’s concern for theodicy, how does this construction preserve God’s justice?

Responding to the second question is relatively straightforward. As God’s promises of reward and punishment are reframed as applying to the righteous collective in the eschaton,^106^ and as the final deserts, which are particular to the righteous and the wicked, are described as befitting their respective moral quality, God’s justice is ultimately not impugned. God keeps his promises to Israel and deals with people according to the moral quality of their lives.

Regarding the first question, from the transtemporal context that views history from beginning to end, which is already known and has now been revealed to Ezra, salvation is already effected for “the righteous.” The end tally of the righteous has already been determined. Those following Ezra’s conversion and transformation in the phenomenological present would not alter this tally. Similarly, this tally would remain unchanged even at the appearance of apostate individuals who forsake the covenant. In this sense, individual action or choice does not determine salvation.

But, for both the author and their readers, life must be lived in the phenomenological sphere of the present, where history is still being worked out toward its *telos*. In the phenomenological sphere, individual action or choice is the only means by which one may distinguish to whom God’s promise of salvation obtains. It is the only means by which one’s individual destiny may be accurately and securely connected to collective destiny. So too, it is the only means by which one may identify their real suffering as meaningful, relating it to the kind of suffering through which “the righteous” must struggle in the attainment of history’s *telos*, their consolation, and thus, the *maius saeculum*. In this sense, while individual action or choice may not determine salvation when considered in the context of the transtemporal sphere, it remains a necessary marker of identity for the phenomenological present. In the end, it is only “the righteous” or “the wise” (cf. 12:38) who are saved; “the righteous” who are revealed as such through their understanding, fidelity, and obedience. That the author employs language particular to the phenomenological present to emphasize the importance of being righteous in conjunction with particular actions, choices, or qualities is thus no surprise; nor does it entail contradiction with the larger framework of apocalyptic determinism.

This suggests that, in apocalyptic determinism, individual action is more important for its symbolic significance than, perhaps, its “real” moral quality.^107^ Or rather, the symbolic apocalyptic framework of the community renders individual action significant. This becomes clearer as one considers individual action at the intersection of the transtemporal imaginary and the phenomenological present. In *4 Ezra*, the collective category of “the righteous” makes sense of individual action, such that individual action can be qualified as righteous. Righteousness or fidelity in *4 Ezra* is rendered meaningful by its connection to “the righteous” and their transtemporal destiny. Absent this connection, “the righteousness” of laboring against the evil heart is not clearly seen, especially in light of present suffering where “unrighteousness” might provide relief. In other words, the transtemporal imaginary, for which apocalyptic determinism provides a structural coherence, makes sense of individual action in the phenomenological present.

In this context, terms like “the righteous,” “the wise,” or “the law” function as symbols that transcend their literal meaning. Indeed, it is precisely in this sense that Carol Newsom articulates the “socially multiaccentual” nature of terms and their function as ideological signs:

In second century Judaism terms such as “Torah,” “Israel,” “covenant,” “righteousness,” “what is good in his eyes,” and many others were precisely the sort of terms that became ideological signs. But as each group used those terms they did so with a different “accentuation.” “Torah” has a different flavor in the Maccabean slogan than it does when the Qumran community speaks of “those who do Torah” . . . Simply put, every ideological sign is the site of intersecting accents. It is “socially multiaccentual.”^108^

In this sense, then, the transtemporal imaginary of *4 Ezra* renders individual action meaningful by interpreting it and enclosing it within a symbolic loop.^109^ Terms like “the righteous,” “Israel,” or “the covenant” become ideological signifiers for the community,^110^ which is itself conceptually bounded by apocalypticism’s notional architecture. That is, “the wise” are those who adhere specifically to Ezra’s conceptual framework in contrast to alternative frameworks like the DP.^111^ This is particularly apparent in the symbolic setting of *4 Ezra* and its transformation of the sense of Torah. Najman notes the curiosity of the narrative’s setting, where the Second Temple is envisioned in the terms of the first temple’s destruction, and the Second Temple’s very existence is effaced.^112^ As Najman proposes, it is an imagining of an “alternate past,”^113^ but this alternate past is used in service to a transformation of the community’s symbols. In the argument of *4 Ezra*, the covenant is no longer, and actually never was, conceived in terms of the DP. Rather, it is contextualized by apocalypticism. So, too, the destruction of the scriptures that coincided with this reimagining, necessitating their recreation and re-textualization at the end of the book, also involves the transformation of the Torah.^114^ In this imagery, the argument involves a disassociation of the Mosaic Torah from the DP, and its recalibration to apocalypticism, where the real message of apocalypticism, the true understanding of covenant and Torah, is entrusted in secret only to the wise.^115^ Here, in terms of the work’s implied community, the Torah becomes a symbol of Ezra’s apocalyptic expression as a whole.

To close the symbolic loop, where individual action is perceived and affirmed to be meaningful within this symbolic structure, the conceptual boundaries of the community are also strengthened and reinforced. In this way, individual action is both interpreted by and reinforces the community’s conceptual boundaries such that individual identity with the collective is both signified and validated. At this intersection, one may pinpoint the conceptual efficacy and importance of individual action. Individual action is a performative symbol of collective identity beyond its “real” moral quality. Action is perceived as “moral” through the apocalyptic framework. As the individual participates in this framework through “moral” action, both their membership in the community is affirmed and the community’s conceptual boundaries are strengthened.

## Conclusion

In this examination of Ezra’s pre- and post-conversion modes of deterministic thinking, I am arguing that the consolation that the author offers to their readers is not simply the prospect of an existential experience where rational avenues otherwise face an impasse. Rather, the consolation that Ezra experiences is both rational and existential. Moreover, the rational sets the stage for the existential in the narrative. Although modern readers encounter tension as the author articulates apocalyptic determinism on the one hand and human moral responsibility on the other hand, an underlying coherence underlies both elements. Indeed, the consolation the author offers their readers may be more, but it is certainly nothing less than a potential resolution to the cognitive dissonance that the destruction of the Second Temple represented with respect to God’s justice, goodness, and promises to Israel.

Lorenzo DiTommaso argues that “*4 Ezra* aims to console its intended readers in view of the catastrophic loss by assuring them that salvation is imminent, justice remains operative, and existence still has a purpose.”^116^ The author’s articulation of apocalyptic determinism is essential to this aim. On the one hand, it allows the author to coherently affirm that God is just in relation to his promises and that salvation is certain for the righteous. By dramatizing Ezra’s distress, the author has found a way to express both their problems with the DP and the answers that a deterministic apocalyptic historiography can provide.^117^ In this way, the author demonstrates apocalypticism’s internal logic, providing coherence in lieu of (in the author’s view) incoherence such that grief finds consolation. On the other hand, the author’s notion of human moral agency underscores the necessity and importance of individual action to participate in a collective righteous destiny. For the text’s implied community, this not only provides a manner by which the community’s boundaries are defined and reinforced, but it also offers a system of self-validation. Where members of the community continue to affirm this apocalyptic historiography, and where new members join this framework, the framework’s inherent truth is validated to the community. Even where it is rejected or resisted, once again, the intrinsic truth of the framework is validated to the community.

Patrick Tiller wrote that “the text of *4 Ezra* represents apocalyptic concession to the failure of an apocalyptic worldview.”^118^ He states that “in the end, Pseudo-Ezra can only affirm God’s goodness and justice, but he cannot understand it; thus, he leaves his readers unsatisfied.”^119^ In contrast to this view, I hope to have shown how a rational coherence underlies the author’s articulation of apocalyptic determinism and human responsibility. Indeed, this aspect is essential to the author’s goal of conversion. That it results only in the salvation of a righteous few is no small boon where, in the author’s view, the alternative is comprehensive ruin.

## Notes

1. Michael E. Stone, *Fourth Ezra: A Commentary on the Book of Fourth Ezra* (Minneapolis: Fortress Press, 1990), 32.

2. Earl Breech, building on Hermann Gunkel before him, proposed something similar: “when the people come out of the field to beg Ezra not to desert them, he replies that he has been away from them ‘to pray on account of the desolation of Zion and to seek mercy on account of the humiliation of your sanctuary’ (12:48). It is clear, then, that Ezra’s questionings serve to express the religious problematic which the community experiences” (“These Fragments I Have Shored Against My Ruins: The Form and Function of *4 Ezra*,” *JBL* 92 [1973]: 267–74, 271). By extension, the comfort, and perhaps the manner in which Ezra receives such comfort, is also intended for the community. Indeed, Breech concludes that “the author, under the stress of the consciousness that Jerusalem had once again been wasted, took up the broken fragments of the community’s traditions and, drawing upon ancient patterns of consolation, constructed a literary work in a form or a pattern that might dispel the community’s religious confusion” (“These Fragments I Have Shored Against My Ruins,” 274).

3. Stone, *Fourth Ezra*, 32. Stone argues that the following conceptual touchpoints are affirmed by the author: “God’s workings are a mystery and beyond human comprehension; God loves Israel and will vindicate Israel in the end; God rejoices over the few saved and is not concerned over the many damned; God’s mercy works in this world, while his justice is fully active only in the world to come” (Stone, *Fourth Ezra*, 36).

4. Bruce W. Longenecker, “Locating *4 Ezra*: A Consideration of Its Social Setting and Functions,” *JSJ* 28 (1997): 271–93, 286. Loren T. Stuckenbruck also highlights the way in which Ezra’s grief is emphasized in the text (cf. 10:39). It seems to me, however, that he overlooks a major aspect of Ezra’s conversion when he writes that Ezra is characterized all along by “his sincere lament and grief at the destruction of Jerusalem (which extended to his attempt to console the woman in mourning), on the one hand, *and the anticipation of the eschatological moment when God’s purposes for Israel will be realized*” (“Ezra’s Vision of the Lady: The Form and Function of a Turning Point,” in *Fourth Ezra and Second Baruch: Reconstruction After the Fall*, ed. Matthias Henze and Gabriele Boccaccini [Leiden; Boston: Brill, 2013], 137–50, 149; emphasis mine). The “eschatological moment” is something which Ezra comes to adopt through the process of dialogue with the angel. It is not affirmed by Ezra all along.

5. Karina Martin Hogan, *Theologies in Conflict in 4 Ezra: Wisdom Debate and the Apocalyptic Solution* (Leiden: Brill, 2008), 40. John J. Collins follows Hogan in viewing *4 Ezra* in terms of a three-way debate (“The Idea of Election in 4 Ezra,” *JSQ* 16 [2009]: 83–96.

6. Lorenzo DiTommaso, “Who Is the ‘I’ of *4 Ezra*?” in *Fourth and Second Baruch: Reconstruction after the Fall*, ed. Matthias Henze and Gabriele Boccaccini (Leiden: Boston: Brill, 2013), 121–46, 130.

7. DiTommaso, *4 Ezra*, 133.

8. Hindy Najman, *Losing the Temple and Recovering the Future: An Analysis of 4 Ezra* (New York: Cambridge University Press, 2014), 158.

9. Najman, *Losing the Temple*, 158.

10. Najman, *Losing the Temple*, 158.

11. Gore-Jones, *When Judaism Lost the Temple: Crisis and Response in 4 Ezra and 2 Baruch* (Turnout: Brepols, 2020), 63.

12. Gore-Jones, *When Judaism Lost the Temple*, 63.

13. Gore-Jones, *When Judaism Lost the Temple*, 63. Gore-Jones also writes that “for the author, faithfulness to the Mosaic Torah is key for salvation . . . not only did he not abandon the Mosaic tradition, but he also endeavoured to incorporate his new worldview into the old” (*When Judaism Lost the Temple*, 63). Longenecker’s comments regarding covenantal thought in Early Judaism are relevant here. “Although the covenant is fundamental to much of the literature of Early Judaism, those texts themselves suggest that two central components of covenantalism—the law and the people of Israel—are subject to (re)definition. If we extract these two components from particular texts in order to notice common themes and the like, we must also recognize that what we are left with is something quite bare-boned which tends to be an ahistorical abstraction. As soon as flesh is given to this ‘skeleton’, what becomes visible is not so much that which was common to the various groups which comprised Early Judaism but, instead, their idiosyncratic definition of that commonplace” (*Eschatology*, 31–32). In this light, and particularly because Gore-Jones herself highlights how the sense of “Torah” is transformed in *4 Ezra*, her reference to the “Mosaic Torah” seems a bit odd. The use of the “Torah,” itself somewhat problematic as a term in *4 Ezra* (cf. Karina Martin Hogan, “The Meanings of Tôrâ in *4 Ezra*,” *JSJ* 38 [2007]: 530–52, 534), cannot be simply equated to the “Mosaic Torah” without further nuance. It would be more accurate to state that *4 Ezra* transforms the sense of “Mosaic Torah” such that the work’s eschatological visions are part and parcel to it. The old is transformed into something different as it is integrated into the new.

14. Albert Thompson, *Responsibility for Evil in the Theodicy of IV Ezra: A Study Illustrating the Significance of Form and Structure for the Meaning of the Book* (Missoula: Scholars Press, 1977), 339–40.

15. Thompson, *Responsibility for Evil*, 340–42. Similarly, James H. Charlesworth argues explicitly that “if one author has compiled the heterogeneous traditions gathered, he has not presented a synthesis that leads to a coherent position. The harsh questions are never adequately answered” (“*4 Ezra* and *2 Baruch*: Archaeology and Elusive Answers to Our Perennial Questions,” in *Interpreting 4 Ezra and 2 Baruch: International Studies*, ed. Gabriele Boccaccini and Jason M. Zurawski [New York: Bloomsbury, 2014], 155–72, 165).

16. Hogan, *Theologies*, 9.

17. Dereck Daschke, *City of Ruins: Mourning the Destruction of Jerusalem Through Jewish Apocalypse* (Leiden: Brill, 2010), 103–39, 106.

18. Daschke, *City of Ruins*, 109.

19. Longenecker, “Locating *4 Ezra*,” 286.

20. Cf. Longenecker, “Locating *4 Ezra*,” 287.

21. Elsewhere, Longenecker writes that the two sides of the debate in the dialogue sections “are so mismatched that their incompatibility precludes real dialogue and progress. But perhaps there is method in the author’s apparent madness. He sustains a dialogue which has the appearance of a potentially productive debate but which never develops into much of anything, the purpose being to provoke in the audience an emotional response; the longer the dialogue remains at a standstill, the more the reader is to be drawn into Ezra’s own frustration” (Bruce Longenecker, *Eschatology and the Covenant: A Comparison of 4 Ezra and Romans 1-11* [Sheffield: JSOT Press, 1991], 94).

22. Najman, *Losing the Temple*, 157–58.

23. Katell Berthelot, “Is God Unfair? The Fourth Book of Ezra as a Response to the Crisis of 70 C. E.,” in *Judaism and Crisis. Crisis as a Catalyst in Jewish Cultural History*, ed. Armin Lange, Diethard Römheld, and Matthias Weigold (Göttingen: Vandenhoeck & Ruprecht, 2011), 73–89, 82.

24. Gore-Jones, *When Judaism Lost the Temple*, 99.

25. DiTommaso, “*4 Ezra.*”

26. George W. E. Nickelsburg, “Torah and the Deuteronomic Scheme in the Apocrypha and Pseudepigrapha: Variations on a Theme and Some Noteworthy Examples of Its Absence,” in *Das Gesetz im frühen Judentum und im Neuen Testament: Festschrift für Christoph Burchard zum 75. Geburtstag*, ed. Dieter Sänger and Matthias Konradt (Göttingen: Vandenhoeck & Ruprecht; Fribourg: Academic Press, 2006), 222–35.

27. R. J. Coggins and Michael A. Knibb, *The First and Second Books of Esdras* (London: Cambridge University Press, 1979), 245–46.

28. Stone, *Fourth Ezra*, 65.

29. Stone, *Fourth Ezra*, 70. It seems clear that the expressions “free will” and “freedom of choice,” as utilized by Stone and others, rightly intend some notion of compatibilism, in that the author of *4 Ezra* understands human moral agency to be compatible with historical determinism. However, the terms “free will” or “freedom of choice” are problematic for two reasons. First, the term “free will” is commonly associated with philosophical notions of human agency that are incompatible with determinism. In this use, the term suggests an anthropology that is foreign to the text and is potentially anachronistic. Second, since Stone never actually defines “free will” as it applies to *4 Ezra*, the expression does not explain the way in which apocalypticism represents a solution to Ezra’s turmoil regarding determinism and the fate of the wicked.

30. Hogan, *Theologies*, 148.

31. Hogan, *Theologies*, 149.

32. Jonathan Moo, *Creation, Nature and Hope in 4 Ezra* (Göttingen: Vandenhoeck & Ruprecht, 2011), 91.

33. Najman, *Losing the Temple*, 83.

34. Najman, *Losing the Temple*, 84.

35. Cf. Stone, *Fourth Ezra*, 319–20.

36. One might make a distinction between Ezra’s conversion and ultimate transformation at the end of the book. A significant shift does indeed occur in Ezra’s encounter with the weeping woman-city (cf. Stone, *Fourth Ezra*, 319–320; DiTommaso, “*4 Ezra*”). There also seems to be a progressive transformation that continues after this moment until Ezra’s ultimate translation into angelic life (Michael E. Stone, “Seeing and Understanding in *4 Ezra*,” in *Revealed Wisdom: Studies in Apocalyptic in Honour of Christopher Rowland*, ed. John Ashton [Leiden: Brill, 2014], 122–37; Peter-Ben Smit, “Reaching for the Tree of Life: The Role of Eating, Drinking, Fasting, and Symbolic Foodstuffs in *4 Ezra*,” *JSJ* 45 [2014]: 366–87; Meredith J. C. Warren, *Food and Transformation in Ancient Mediterranean Literature* [Atlanta: SBL Press, 2019], 37–58). In writing of Ezra’s “conversion,” then, I reference his internalization of what Uriel has articulated in the dialogues as reflected in his encounter with the weeping woman-city. Ezra’s conversion thus leads to a progressively realized transformation that culminates in his translation into angelic life.

37. “*4 Ezra*,” 130.

38. This necessarily raises the question of where and of how to locate the author’s affirmative views within *4 Ezra*. As mentioned previously, Hogan argues that the apocalypticism of the visions represents a third perspective that resolves, on non-rational grounds, the irresolvable problematic of the work’s initial dialogues, wherein Ezra and Uriel articulate two competing wisdom traditions (*Theologies*). See DiTommaso, “*4 Ezra*,” for a persuasive critique of Hogan’s proposal. I follow both Stone (*Fourth Ezra*) and DiTommaso (“*4 Ezra*”), and those scholars who understand the content of the dialogue and the content of the visions in terms of continuity. The author’s affirmative views, therefore, can be located in the voices of God, Uriel, and the post-conversion Ezra. See Stone, *Fourth Ezra*, 11–23; Hogan, *Theologies*, 9–40; Juan Carlos Ossandón Widow, *The Origins of the Canon of the Hebrew Bible: An Analysis of Josephus and 4 Ezra* (Leiden; Boston: Brill, 2018), 101–5; and Gore-Jones, *When Judaism Lost the Temple*, 24–35 for competing views on the composition and authorial unity of *4 Ezra*.

39. Nickelsburg, “Torah,” 222.

40. That the DP functions as a historiography in contradistinction to apocalypticism, see the treatment of Daniel 9 in John J. Collins, *Daniel: A Commentary on the Book of Daniel* (Minneapolis: Fortress Press, 1993), 360; and Lorenzo DiTommaso, “4QPseudo-Daniel^a-b^ (4Q243–4Q244) and the Book of Daniel,” *DSD* 12 (2005): 101–33, 120–26; and DiTommaso, “Time and History in Ancient Jewish and Christian Writings,” in *Dreams, Visions, and Imaginations: Jewish, Christian, and Gnostic Views of the World to Come*, ed. Jens Schröter, Tobias Nikolas, and Armand Puig i Tàrech (Berlin: De Gruyter, 2021), 53–88, 63–65). For an alternative view, see Gabriele Boccaccini, *Roots of Rabbinic Judaism: An Intellectual History from Ezekiel to Daniel* (Grand Rapids: Eerdmans, 2022), 181–88. See DiTommaso, “4QPseudo-Daniel^a-b^,” 120–26, for a detailed analysis of Boccaccini’s proposals. Of *4 Ezra* specifically, DiTommaso defines the operating principle of Deuteronomic thought in *4 Ezra* as “the conviction that the choices one makes in the present will affect one’s future state of being. These propositions imply a certain theology of history, which in the world of ancient Judaism translates into an understanding of God’s historical relationship with humanity and Israel in particular” (“*4 Ezra*,” 127–28).

41. “The pattern . . . is drawn from Deuteronomy 28–32. Within the structure of the Sinaitic covenant, God lays out the stipulations of the Torah. Obedience brings blessings of the covenant and disobedience its curses. When Israel repents of its sins, the curses are removed and divine blessing returns to Israel” (Nickelsburg, “Torah,” 222). Elsewhere, Nickelsburg provides a more complete definition of the biblical notion of covenant. “The God who lays commandments and obligations upon Israel first chose Israel from among the nations and invited this people into a special relationship . . . Within this relationship Israel’s God presented commandments and ordinances that Israel was to observe faithfully. To the extent that Israel was faithful to its obligations, the people would experience God’s blessings—long life, health and safety, and fertility in one’s family and on the land (Deut 28:1–14). Disobedience, on the other hand, would bring the curses of the covenant upon the people—a shortened life, sickness, drought, famine and barrenness, invasion, and captivity (28:15–29). The cause-and-effect relationship of disobedience and curse could be broken, however. If the nation repented and turned to God—that is, if they began rightly to obey the Torah—they would experience the covenantal blessings (30:1–10)” (*Ancient Judaism and Christian Origins: Diversity, Continuity, and Transformation* [Minneapolis: Fortress Press, 2003], 32). This is similar to E.P. Sanders’s articulation of covenantal nomism (*Paul and Palestinian Judaism: A Comparison of Patterns of Religion* [Minneapolis: Fortress Press, 1977], 422; cf. Nickelsburg, *Ancient Judaism*, 209 n.10). It is no accident that, in Sanders’ analysis, *4 Ezra* articulates a view which deviates from covenantal nomism (*Paul*, 409). It is precisely the DP which is under critique in *4 Ezra*.

42. See Michael E. Stone, “Apocalyptic Literature,” in *Jewish Writings of the Second Temple Period: Apocrypha, Pseudepigrapha, Qumran Sectarian Writings, Philo, Josephus*, ed. Michael E. Stone (Assen, The Netherlands: Van Gorcum; Fortress Press, 1984), 383–442, 384.

43. Cf. John J. Collins, “Apocalypticism as a Worldview in Ancient Judaism and Christianity,” in *The Cambridge Companion to Apocalyptic Literature*, ed. Colin McAllister (New York: Cambridge University Press, 2020), 19–35, 22.

44. Bernard Berofsky, *Determinism* (Princeton: Princeton University Press, 2015 [1971]), 5.

45. Mladen Popović defines (causal) determinism as “everything that happens in time and space is fixed or determined by a necessary chain of causation” (“Apocalyptic Determinism,” *The Oxford Handbook of Apocalyptic Literature*, ed. John. J. Collins [New York: Oxford University Press, 2014], 255–70, 255). He notes that in the history of religions, divine determinism entails an added assumption that “everything is determined by a divine creator, or by fate” (“Apocalyptic Determinism,” 255). But one need not take a position on a particular form of causality to deny a real possibility for alternate states of affairs. This denial is more basic than the affirmation of a particular mode of causality.

46. See Stone, *Fourth Ezra*, 35–36; DiTommaso, “*4 Ezra*,” 128–29; Berthelot, “Is God Unfair?” 80–81 (*contra* Thompson, *Responsibility for Evil*); Longenecker, *Eschatology*, 63–64; and Gore-Jones, *When Judaism Lost the Temple*, 10, 32.

47. See Stone, *Fourth Ezra*, 63, for his summary of the way in which Ezra questions God’s justice in this first discourse.

48. Cf. Stone, *4 Ezra*, 76.

49. Cf. DiTommaso, “*4 Ezra*,” 127–28.

50. See Stone, *Fourth Ezra*, 63–67, for his excursus on the “evil heart.” John Levison argues that the relation between Adam’s evil heart and the evil heart is merely correspondence (*Portraits of Adam in Early Judaism* [Sheffield: JSOT, 1988], 116). Jason M. Zurawski affirms this view (“Rethinking the Divide between *4 Ezra* and *2 Baruch*: Getting to the (Evil) Heart of the Matter,” in *Wisdom Poured Out Like Water*, ed. J. Harold Ellens, Isaac W. Oliver, Jason von Ehrenkrook, James Waddell, and Jason M. Zurawski [Berlin: De Gruyter, 2018], 177–92, 181). De Long, however, notes that the author connects the evil heart from Adam to his descendants with genealogical terms: *nascor, multiplico, semen*, and *filius* (“Ask a Woman,” 120 n8). This language suggests more than simple correspondence. Thus, Stone describes 7:118 as “a restatement of what the author said in 3:20–22, which speaks of the sin of Adam affecting all of his descendants, ‘the disease’ becoming permanent and the ‘evil root’ being in the hearts of humans” (*Fourth Ezra*, 258). Of course, an assessment of the author’s affirmative views on the matter must look beyond Ezra’s pre-conversion articulations.

51. See 7:118–126. In this passage, Ezra has accepted Uriel’s account of God’s promises as referring to a coming transtemporal, eschatological horizon. Stone comments that here, “the ‘immortal age’ is the age to come in which ‘corruption will be overcome’ (6:28)” (*Fourth Ezra*, 259). The dynamic of the DP, however, where human agency is the driving mechanism of history, is still in force. It is not until the vision of the woman-city that Ezra fully adopts the apocalyptic outlook.

52. *in ueritate enim nemo de genitis est qui non impie gessit et de confitentibus qui non deliquit in hoc enim adnuntiabitur iustitia tua et bonitas tua Domine cum misertus fueris eis qui non habent substantiam operum bonorum.* For English citations, the NRSV is cited unless I indicate otherwise. Citations of the Latin text refer to the *Vulgate* (R. Weber. and R. Gryson, eds., *Biblia Sacra Iuxta Vulgatam* [Latin Vulgate-Tagged VULG-T], 5th Edition [Morphologically tagged by Marco V. Fabbri. 2015. Pontificia Università della S. Croce: Rome], version 3.8: Accordance Bible Software [Stuttgart: Deutsche Bibelgesellschaft, 2007]).

53. Carol Newsom, *The Spirit Within Me: Self and Agency in Ancient Israel and Second Temple Judaism* (New Haven: Yale University Press, 2021), 98.

54. Miryam Brand, *Evil Within and Without: The Source of Sin and Its Nature as Portrayed in Second Temple Literature* (Göttingen: Vandenhoeck & Ruprecht, 2013), 133.

55. Longenecker, *Eschatology*, 98. For his proposal of common threads between *1 Enoch*, Paul’s thought, and *4 Ezra* regarding the notion of humanity’s inability with reference to the Torah, see Gabriele Boccaccini, “The Evilness of Human Nature in *1 Enoch*, *Jubilees*, Paul, and *4 Ezra*: A Second Temple Jewish Debate,” in *Fourth Ezra and Second Baruch: Reconstruction After the Fall*, ed. Matthias Henze and Gabriele Boccaccini (Leiden: Brill, 2013), 63–79.

56. Cf. Berthelot, “Is God Unfair?” 76.

57. This is distinct from other heuristic approaches. John J. Collins advocates primarily for understanding apocalypses in terms of literary genre (John J. Collins, ed., *Apocalypse: The Morphology of a Genre* [*Semeia* 14; Missoula: Scholars Press, 1979]; cf. John J. Collins, *The Apocalyptic Imagination*, 3rd ed. [Grand Rapids: Eerdmans, 2016]). He also affirms, however, that this genre entails a distinct worldview (See Collins, “Apocalypticism as a Worldview”). One prominent and influential alternative to Collins’ genre approach has been the definition of “apocalyptic” proposed by Christopher Rowland. Rowland offers an essentialist definition that focuses on the concept of revelation. “Apocalyptic seems essentially to be about the revelation of the divine mysteries through visions or some other form of immediate disclosure of heavenly truths” (Christopher Rowland, *The Open Heaven: A Study of Apocalyptic in Judaism and Early Christianity* [New York: Crossroad, 1982], 70.

58. DiTommaso, “Time and History,” 56.

59. Lorenzo DiTommaso, “The Apocalyptic Other,” in *The “Other” in Second Temple Judaism: Essays in Honour of John J. Collins*, eds. Daniel C. Harlow *et al.* (Grand Rapids: Eerdmans, 2011), 221–46, 221. Cf. DiTommaso, “The Apocrypha and Apocalypticism” in *The Oxford Handbook of the Apocrypha*, ed. Gerbern Oegema (New York: Oxford University Press, 2021), 219–52, 222–23.

60. DiTommaso, “Time and History,” 60. See also John J. Collins, “Apocalyptic Eschatology as the Transcendence of Death,” *CBQ* 36 (1974): 21–43. He argues that apocalyptic eschatology is distinguished from prophetic eschatology by its emphasis on an individual transcendence of death (cf. Collins, “Apocalypticism as a Worldview,” 29–31). Note also Michael E. Stone, “Three Transformations in Judaism: Scripture, History, and Redemption,” *Numen* 32 (1985): 218–35, for his discussion of the development of history and meta-history within Judaism.

61. DiTommaso, “Time and History,” 60.

62. DiTommaso, “Time and History,” 61.

63. DiTommaso, “Time and History,” 62.

64. Cf. Collins, “Apocalypticism as a Worldview,” 29–31; DiTommaso, “Time and History,” 61–62.

65. “Apocalyptic eschatology is permeated by the expectation of the imminent end and, for it, the advent of the end does not depend upon human action” (Stone, “Apocalyptic Literature,” *Jewish Writings of the Second Temple Period: Apocrypha, Pseudepigrapha, Qumran Sectarian Writings, Philo, Josephus*, ed. Michael E. Stone [Assen: Van Gorcum; Philadelphia: Fortress Press, 1984], 383–442, 383).

66. DiTommaso, “Time and History,” 61–62.

67. Collins, “Apocalypticism as a Worldview,” 22.

68. *ecce dies ueniunt* . . . cf. 5:1; 12:13.

69. This is specifically in reference to the Latin text of the *Vulgate.* Of course, the most natural way to refer to the future is through the future indicative. The point, however, is that in the way in which the future is described, no alternatives are considered possible.

70. See Michael E. Stone, “Coherence and Inconsistency in the Apocalypses: The Case of ‘The End’ in *4 Ezra*,” *JBL* 102 (1983): 229–43, for the meaning of “the end” in *4 Ezra.* “The analysis of the term ‘the end’ (*finis* and equivalents) showed a number of options at play. From its use in the book, it was concluded to denote ‘the crucial turning point of the eschatological process.’ The exact identification of that point, where such existed, was determined firstly by context and purpose, and secondly by the associational complex involved” (p. 243). The term’s coherency as defined by Stone underscores the temporal dualism of apocalyptic eschatology, where the idea of a distinct break between the present and the future is inherent to its use.

71. Cf. DiTommaso, “*4 Ezra*,” 127.

72. “Ezra does still question the angel, but his questions are intended merely to elicit information, not to challenge what the angel says” (Knibb, *Second Esdras*, 109).

73. . . . et *qui super terram inhabitant quae sunt super terram intellegere solummodo possunt et qui super caelos super altitudinem caelorum*. Cf. DiTommaso, “*4 Ezra*,” 128.

74. Specific elements of the predetermined future include the end of Zion’s humiliation (6:19); a transformation in the heart for those who will be saved (6:26); the revelation of the Messiah with his entourage, the period of his dwelling on earth, and his death (7:28–29); the nature of post-mortem existence for both the righteous and the wicked (7:78–101); the rise of Rome, its fall with the coming of the Messiah, and the deliverance of the remnant until the day of judgement (12:13–36); a final conflict between the wicked nations and the Messiah, who defeats them such that both Zion and the righteous are preserved (13:21–36); and the gathering of the lost tribes of Israel (13:37–53).

75. Cf. Popović, “Apocalyptic Determinism,” 259–60.

76.
*quantum enim tempus ex quo longanimitatem habuit Altissimus his qui inhabitant saeculum et non propter eos sed propter ea quae prouidit tempora.*

77. *quemadmodum enim infans non parit nec ea quae senuit adhuc sic ego disposui a me creatum saeculum* (5:49).

78. *tunc cogitaui et facta sunt haec per me solum et non per alium ut et finis per me et non per alium* (6:6).

79. Cf. Stone, *Fourth Ezra*, 155.

80. Uriel affirms that God has made the *saeculum* for Israel. But Uriel also affirms that the creation was judged after Adam’s transgression (*propter eos enim feci saeculum et quando transgressus est Adam constitutiones meas iudicatum est quod factum est*; 7:11). In this way, Uriel could be suggesting that God changed his plan, along with the nature of the world, in response to Adam’s sin. However, given the emphatic statement about God’s plans for judgment which were in effect prior to creation at 7:70, as well as certain ambiguities in the text itself, two other options seem preferable. Uriel’s reference to the *saeculum* which was created for Israel is rendered ambiguous by the way he goes on to contrast the entrances of *huius saeculi* (7:12) and the entrances of *maioris saeculi* (7:13). Because the author does not initially specify which *saeculum* pertains to Israel, one could understand it to refer to either “this world” or the “greater world.” If one opts for the latter, God has not altered his plan by judging the creation for Adam’s transgression. If one opts for the former, given that Uriel goes on to state that judgement was planned before creation, one might nuance the sense in which this *saeculum* was created for Israel. It was created for Israel as a trial for receiving the *maius saeculum* (cf. 7:127; 8:2). This second option seems more likely given the comparison that Uriel makes between the heir of a city who comes into their inheritance by passing through its narrow gates, such that Uriel states “*sic est et Israhel pars*” (7:10). Moreover, this reading is underscored by 7:74, where God’s patience with humanity is not for humanity’s sake, but “because of the time that he has foreordained” (*quantum enim tempus ex quo longanimitatem habuit altissimus his qui inhabitant saeculum et non propter eos sed propter ea quae prouidit tempora*). Stone (*Fourth Ezra*, 234) comments that “what seems like a delay of the eschaton caused by divine long-suffering is in fact only the predetermined order of events. The result of this is to set long-suffering itself into the predetermined divine plan” (*Fourth Ezra*, 234; cf. 299; his comments on 9:20 are also relevant). Jason Zurwaski contends that at 7:10–16, the world to come is the one which was promised to Israel. He argues that where the Latin text reads “*quod factum est*,” it should be translated as “that which happened” as opposed to “that which was made” (“The Two Worlds and Adam’s Sin: The Problem of *4 Ezra* 7:10–14,” in *Interpreting 4 Ezra and 2 Baruch*, ed. Gabriele Boccaccini and Jason. M. Zurawski [New York: Bloomsbury T&T Clark, 2014], 97–106, 102–4). Zurawski also argues that the causal sense of *4 Ezra* 7:12 refers back to the first part of 7:11 where God made the world (to come) for Israel’s sake, and not back to Adam’s transgression.

81. *non fecit Altissimus unum saeculum sed duo*.

82. Stone, *Fourth Ezra*, 234.

83. Cf. Stone, “Apocalyptic Literature.”

84. *tunc cogitaui et facta sunt haec per me solum et non per alium* (6:1–6).

85. *ut et finis per me et non per alium* (6:6).

86. God’s agency in bringing about “the end” is also emphasized through the use of personal pronouns. So, for example, the Messiah is referred as “my Messiah” (7:28). Furthermore, it is rendered explicit in passages such as 9:2, where the end is characterized as God’s visitation to his creation. Additionally, 7:78 describes death as one area of God’s jurisdiction such that God sends out a decree concerning the time in which a person must die. The image here is of God as a governor whose jurisdiction extends even to authorizing individual deaths; something from which there is no process of appeal.

87. Cf. Stone, “Seeing and Understanding,” 128.

88. Reward and punishment are distinctively post-mortem or transtemporal realities in 7:31–44.

89. The post-mortem, or transtemporal, realities of reward and punishment are depicted as applying to individuals. So, for example, Uriel states that “no one shall ever pray for another on that day, neither shall anyone lay a burden on another; for then all shall bear their own righteousness and unrighteousness” (7:105). However, human destiny is primarily constructed in collective terms such that the number of righteous souls has been predetermined (4:35–36). Individual choice does not affect the number of righteous souls which have been predetermined. Thus, individual destiny concerns an individual’s identification with a greater collective such that individual destiny is necessarily connected to and is referential of collective destiny. See DiTommaso, “*4 Ezra*,” 122–23.

90. “Ezra’s challenge to God in 3:4–36 . . . sets forth the issue that focuses the central concerns of the whole book, viz. the fate of Israel and the destruction of Zion, and the book may be regarded as a series of responses to it. It should be stressed that the problem of concern to the seer is the sufferings of Zion, while the more general issue of Adam’s sin and its results is actually part of the seer’s argument and is adduced as a way of heightening this central point. The particular dimension of this theme stressed in the present passage is the questioning of the destruction of Zion in terms of providence and divine justice” (Stone, *Fourth Ezra*, 61).

91. On this point, it is irrelevant whether the figure of Ezra represents the author’s real experience of conversion (cf. Stone, *Fourth Ezra*, 32; DiTommaso, “*4 Ezra*”), or whether this is simply a literary construction reflecting the struggles of a community (cf. Breech, “These Fragments”). In either case, the cognitive dissonance that is expressed requires an adequate response. To be clear, I am not arguing that the existential is inferior to the rational. Rather, as outlined at the beginning of this paper, there is a tendency in scholarship to frame the existential against the rational. By contrast, I contend that Ezra’s resolution is only actualized as the rational and existential function together. In other words, I think that it is unlikely that the existential component on its own can overcome an *ongoing* cognitive dissonance. At the same time, the existential component is necessary for Ezra’s internalization of apocalypticism’s rationality (cf. Stone, *Fourth Ezra*, 32).

92. Cf. Van Inwagen, “Moral Responsibility, Determinism, and the Freedom to do Otherwise,” *The Journal of Ethics* 3 (1999): 341–50. For the variety of philosophical views on compatibilism and related notions of free will, see Kevin Timpe, Meghan Griffith, and Neil Levy, eds., *The Routledge Companion to Free Will* (London: Routledge, 2017). See Bruce Reichenbach, *The Law of Karma: A Philosophical Study* (Basingstoke: Macmillan, 1990), 45 for what, I think, more closely captures the sense of free will in non-technical conceptualizations. This sense seems to correspond well to Stone’s use of the term in his commentary on *4 Ezra* (cf. Stone, *Fourth Ezra*, 288, 297). DiTommaso distinguishes between two different contextual referents for divine will and free will within apocalypticism. “Divine will” concerns “historic events” and “focuses on the preordained fates of groups of people” (“*4 Ezra*,” 123). By contrast, “free will operates on the level of individuals . . . as the decision to persevere in the good or persist in evil” (“*4 Ezra*,” 123).

93. See Van Inwagen, “Moral Responsibility.”

94. “Freedom, Causality, Fatalism and Early Stoic Philosophy,” *Phronesis* 30 (1985): 274–304.

95. “Apocalyptic Determinism.”

96. See also 7:37–8; 7:127–131; 8:55–62; 9:17–22.

97.
*qui ergo commorantes sunt in terra hinc cruciabuntur quoniam sensum habentes iniquitatem fecerunt et mandata accipientes non seruauerunt ea et legem consecuti fraudauerunt eam quam acceperunt . . .*

98. *Libertas* is employed in few places which reference to moral agency (8:56; 9:11–12). In my view, these references to *libertas* are contrasted specifically to *cruciatus*. Because torture is an act of subjugation and a means of external control (cf. 9:11–12), the author’s invocation of *libertas* remains closely resonant of its meaning as political freedom (cf. 2 Macc 7:1, where *cruciatus* is specifically identified as means of compulsion [*conpello*]). See Chaim Wirszubski, *Libertas as a Political Idea at Rome During the Late Republic and Early Principate* (London: Cambridge University Press, 1960), 1, 7–8, and Mason Hammond, “Res Olim Dissociabiles: Principatus Ac Libertas: Liberty Under the Early Roman Empire,” *Harvard Studies in Classical Philology* 67 (1963): 93–113, for *libertas* as political freedom.

99. “These words, as also those of verse 22, imply that salvation even of the few is only the result of God’s grace” (Knibb, *Second Esdras*, 217). “Previously it had been stressed that the wicked determined their own fate and God’s concern is for the righteous. Here the same point is subtly made by the formulation of the verbs of creation. While the multitude that perishes is said to have ‘been born in vain,’ employing a passive verb, of those who are saved God says, ‘*I* have perfected them’” (Stone, *Fourth Ezra*, 301).

100. *Pace* Longenecker, *Eschatology*, 91.

101. It is worth noting here that references to “you” are in the plural form in the *Vulgate* (*uobis*).

102.
*radix signata est a uobis infirmitas extincta est a uobis et mors absconsa est infernum fugit et corruptio in obliuionem transierunt dolores et ostensus est in finem thesaurus inmortalitatis.*

103. Although this notion is articulated by Ezra in his pre-conversion thought, Uriel does not explicitly correct this view. Indeed, it is implicit in 8:53.

104. See Note 92.

105. See Note 92.

106. At points, it is clear that the nation of Israel is still the target of God’s promise of salvation, although it is only applicable to the righteous remnant within the nation (9:8; 12:34; 13:39–49). This idea of the remnant provides a coherent link between collective, national destiny and individual destiny (*pace* Collins “Election;” cf. DiTommaso “*4 Ezra*,” 121–23).

107. It seems to me that, in apocalyptic texts generally, morality is not connected to “goodness” or “righteousness” as abstracted, self-sufficient categories. Rather, they are always socially and symbolically bound to both the texts’ implied community and apocalypticism’s conceptual boundaries.

108. *The Self as Symbolic Space: Constructing Identity and Community at Qumran* (Leiden: Brill, 2004), 10–11.

109. Anthony P. Cohen writes the following about community: “community is . . . a boundary-expressing symbol. As a symbol, it is held in common by its members; but its meaning varies with its members’ unique orientations to it. In the face of this variability of meaning, the consciousness of community has to be kept alive through manipulation of its symbols. The reality and efficacy of the community’s boundary—and, therefore, of the community itself—depends upon its symbolic construction and embellishment” (*Symbolic Construction of Community* [London: Routledge, 1985], 15). So too, “behavior does not ‘contain’ meaning intrinsically, rather, it is found to be meaningful by an act of interpretation: we ‘make sense’ of what we observe” (*Symbolic Construction of Community*, 17). “It is that the community itself and everything within it, conceptual as well as material, has a symbolic dimension, and, further, that this dimension does not exist as some kind of consensus of sentiment. Rather, it exists as something for people ‘to think with’. The symbols of community are mental constructs: they provide people with the means to make meaning. In so doing, they also provide them with the means to express the particular meanings which the community has for them” (*Symbolic Construction of Community*, 19).

110. By community, I refer to the text’s implied community (cf. Breech “These Fragments”).

111. In this way, it is very possible for such a conceptually bounded community to exist in spatial proximity to other Jewish groups. In other words, it is perfectly feasible that the author’s “apocalyptic community” was not physically separated from or spatially distinct to other groups like some sectarian groups could be.

112. *Losing the Temple*, 16–18.

113. *Losing the Temple*, 17.

114. Cf. *Losing the Temple*, 156.

115. Cf. Hogan, “Tôrâ;” Gore-Jones, *When Judaism Lost the Temple*, 102. Where Gore-Jones writes that “*4 Ezra* asserts its authority and worth not by challenging the Mosaic tradition but by grafting itself onto that tradition” (*When Judaism Lost the Temple*, 101), it seems to me that she has underestimated the way that *4 Ezra* critiques the DP and transforms the notion of Torah. It is more accurate to state that *4 Ezra* asserts its own authority and worth by adapting and transforming the Mosaic Torah in accordance with its apocalypticism.

116. DiTommaso, “*4 Ezra*,” 132.

117. Cf. DiTommaso, “*4 Ezra*,” 132–33.

118. “Anti-Apocalyptic Apocalypse,” in *For a Later Generation: The Transformation of Tradition in Israel, Early Judaism, and Early Christianity*, ed. Randal A. Argall, Beverly A. Bow, and Rodney A. Werline (Harrisburg: Trinity Press International, 2000), 258–65, 260.

119. “Anti-Apocalyptic Apocalypse,” 264.

